# Molecular Epidemiology of Clinical Carbapenem-Resistant *Acinetobacter baumannii-calcoaceticus* complex Isolates in Tertiary Care Hospitals in Java and Sulawesi Islands, Indonesia

**DOI:** 10.3390/tropicalmed7100277

**Published:** 2022-09-30

**Authors:** Heriyannis Homenta, Julyadharma Julyadharma, Hani Susianti, Noorhamdani Noorhamdani, Dewi Santosaningsih

**Affiliations:** 1 Doctoral Program in Medical Science, Faculty of Medicine, Universitas Brawijaya, Malang 65145, Indonesia; 2 Department of Clinical Microbiology, Faculty of Medicine, Sam Ratulangi University, Manado 95163, Indonesia; 3 Laboratory of Clinical Microbiology, Prof. Dr. R. D. Kandou Hospital, Manado 95163, Indonesia; 4 Department of Clinical Pathology, Faculty of Medicine, Brawijaya University, Malang 65145, Indonesia; 5 Department of Clinical Pathology, Dr. Saiful Anwar Hospital, Malang 65112, Indonesia; 6 Department of Clinical Microbiology, Faculty of Medicine, Brawijaya University, Malang 65145, Indonesia; 7 Department of Clinical Microbiology, Dr. Saiful Anwar Hospital, Malang 65112, Indonesia

**Keywords:** *A. baumannii-calcoaceticus* complex, carbapenem resistant, Indonesia

## Abstract

Carbapenem-resistant *Acinetobacter baumannii (A. baumannii)-calcoaceticus* complex (CRAb-cc) is an important pathogen causing nosocomial infections worldwide; however, molecular epidemiology of the *A. baumannii-calcoaceticus* complex in Indonesian hospitals is scarce. This study aimed to determine the clonal relatedness of CRAb-cc in two tertiary care hospitals in Malang and Manado in Indonesia. The CRAb-cc isolates from routine clinical cultures in two tertiary care hospitals in Malang and Manado were identified using the Vitek2^®^ system (bioMérieux, Lyon, France). Multi-locus variable-number tandem-repeat analysis (MLVA) typing, multi-locus sequence typing (MLST), clonal complex (CC), and phylogenetic tree analysis were conducted for a subset of isolates. Seventy-three CRAb-cc isolates were collected. The CRAb-cc isolates were frequently found among lower-respiratory-tract specimens. We detected the MLVA type (MT) 1, MT3, and MT4 CRAB-cc isolates belonging to the sequence type (ST) 642, and CC1 was the predominant clone in this study. In conclusion, we identified the clonal relatedness of *A. baumannii-calcoaceticus* complex isolates in two tertiary care hospitals in Malang and Manado in Indonesia. Further study is required to investigate the clinical importance and distribution of ST642 in Indonesian hospitals for developing prevention and control measures.

## 1. Introduction

The World Health Organization includes *Acinetobacter baumannii* (*A. baumannii*) as one of the six ESKAPE organisms. ESKAPE is the abbreviation of *Enterococcus faecium*, *Staphylococcus aureus*, *Klebsiella pneumoniae*, *A. baumannii*, *Pseudomonas aeruginosa*, and *Enterobacter* spp. [1,2]. The ESKAPE pathogens are potentially resistant to antimicrobial agents and associated with nosocomial infections [3].

*A. baumannii* is a Gram-negative bacteria that is ubiquitously found in the environment, including soil and water [4]. However, *A. baumannii* has emerged as the most important healthcare-associated pathogens, which is involved in ventilator-associated pneumonia, urinary tract infections, surgical site infections, and bacteremia [5]. Three related species of the Acinetobacter genus, *A. baumannii*, *Acinetobacter pittii*, and *Acinetobacter nosocomialis*, are often encountered in human specimens and are considered as nosocomial pathogens [4]. They are designated as the *A. baumannii-calcoaceticus* complex due to the phenotypic similarity [1].

Carbapenem is a class of antibiotics that are frequently used for *A. baumannii-calcoaceticus* complex infections, leading to the rapid emergence of carbapenem-resistant *A. baumannii-calcoaceticus* complex (CRAb-cc) [6,7,8]. CRAb-cc infections exacerbate public health problems because of limited antibiotic therapy leading to prolonged hospital stays, immobility, increased morbidity, increased mortality, and additional costs [9]. Furthermore, previous studies reported that the MDRO infections resulted in worse clinical outcomes than susceptible strains [10,11,12]. The Center for Disease Control and Prevention (CDC) reported that *Acinetobacter* spp. Infections included about 8500 cases of nosocomial infection, and as many as 700 such deaths were related to antibiotic resistance [13]. Multiple studies revealed that carbapenem-resistant *A. baumannii* isolates were frequently isolated among patients with pneumonia, followed by patients with bloodstream infections and urinary tract infections, particularly in the intensive care unit (ICU) [14,15,16]. The prevalence of carbapenem-resistant *A. baumannii* varies between 30% in USA and 94% in Greece [16]. A multicenter study in Indonesia reported that the prevalence of carbapenem-resistant *A. baumannii* bloodstream infections increased from 49% in 2016 to 57% in 2018 [17].

The carbapenem-resistant *A. baumannii* are capable of surviving in the hospital environment for the long-term [4,18]; moreover, the cross-transmission to patients may happen [19]. In addition, the selective pressure at the genomic level and gene transfer mediated by highly transmissible plasmids occur while bacteria survive in the hospital [4,20,21]. These events might be associated with the outbreak of carbapenem-resistant *Acinetobacter baumanii*, particularly in the ICU setting [16].

The key mechanism of carbapenem-resistant *A. baumannii* is the ability to produce ß-lactamase enzymes. Oxacillinase (OXA), particularly the OXA-23-like enzyme encoded by *blaOXA-23-like*, is the predominantly ß-lactamase enzyme found in several countries, such as France, Saudi Arabia, China, and Indonesia [19,22,23]. 

Molecular epidemiology studies were important to identify the main clones and epidemiological linkages of CRAb-cc isolates from different regions [24,25]. Several molecular epidemiology studies showed genetic diversities of carbapenem resistant *A. baumannii* clinical isolates among countries [26]; sequence types (ST) 1 and ST2 were found in the USA [27], and CC92 was predominant in Asia [28]. Furthermore, typing of multidrug resistant bacteria is essential to controlling and preventing hospital outbreaks [29]. Pulse field gel electrophoresis (PFGE) is considered as the gold standard to detect the clonality of many pathogenic bacterial isolates. A previous study reported the high discriminatory power of PFGE compared to multi-locus sequence typing (MLST) and multiple locus variable number tandem-repeat analysis (MLVA) for *A. baumannii*; however, PFGE is a more labor-intensive method. On the other hand, MLVA is less expensive and less labor-intensive, though it has some advantages, including high resolution, data portability, and intra-laboratory reproducibility [25]. Moreover, MLST is more reproducible than other genotyping methods employed for surveillance [30].

The clonality study of CRAb-cc in Indonesia is scarce. A single-center study in Jakarta, Indonesia, reported several clones of CRAb-cc, including ST195, ST208, ST218, ST642, and new STs detected among ICU patients [19]. However, another previous study in Surabaya, Indonesia, found ST1000, ST1089, and a new ST that had not been identified [31]. Until now, the predominant clone of CRAB-cc in Indonesian hospitals remains unclear. This study aimed to get further insights into the molecular epidemiology of clinical CRAb-cc isolates in two tertiary care hospitals in Indonesia, including Dr. Saiful Anwar Hospital, in Malang, Java Island, and Prof. Dr. R. D. Kandou Hospital, in Manado, Sulawesi Island.

## 2. Materials and Methods

### 2.1. Setting and Study Design

Two tertiary care hospitals were involved in this study: Dr. Saiful Anwar Hospital in Malang (Java Island; 885 beds) and Prof. Dr. R. D. Kandou Hospital in Manado (Sulawesi Island: 1062 beds). The study was performed from March 2019 to August 2019 in Dr. Saiful Anwar Hospital and from June 2019 to November 2019 in Prof. Dr. R. D. Kandou Hospital [32]. The clinical specimens were collected from patients hospitalized in the respective intensive care units (ICU; 37 specimens), neonatal intensive care units (NICU; 8 specimens), cardiovascular intensive care units (CVICU; 3 specimens), medical wards (21 specimens), and surgical wards (4 specimens) in the two tertiary care hospitals. This study was approved by the medical ethics committees of Dr. Saiful Anwar Hospital (Malang) and Prof. Dr. R. D. Kandou Hospital (Manado) (number 400/059/K.3/302/2019 and number 054/EC-KEPK/IV/2019, respectively). 

### 2.2. Bacterial Isolates

*A. baumannii-calcoaceticus* complex isolates were obtained as a part of routine diagnostic procedure and analyzed anonymously. Only one clinical culture with *A. baumannii-calcoaceticus* complex per patient was included in this study. The strains were isolated and identified from blood, urine, the lower respiratory tract, and wounds based on clinically indicated cultures in each hospital involved in this study. 

The identification and antibiotic susceptibility test of CRAB-cc were performed by the VITEK2^®^ system (VITEK^®^ 2 GN ID and VITEK^®^ 2 AST-GN93; bioMérieux, Lyon, France) [19] and subsequently analyzed according to CLSI 2019 guideline [33]. The CRAB-cc isolates were stored in trypticase soy broth with 10% glycerin and stored at −80 °C until further characterization.

### 2.3. DNA Extraction and Carbapenemase Genes’ Identification

**Figure 1:**
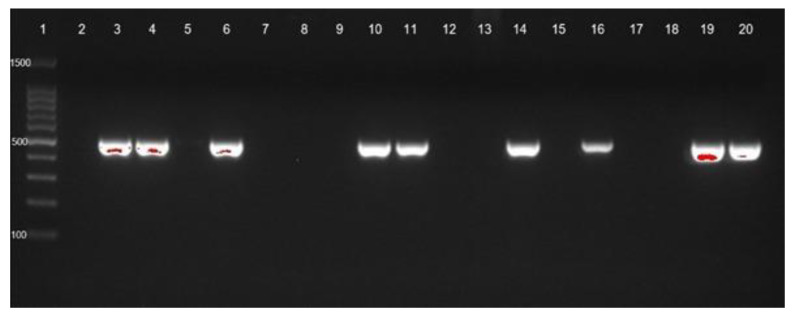
blaOXA-23 gene identification of CRAb-cc isolates from Prof. Dr. R. D. Kandou Hospital, Manado. Lane 1 = DNA ladder; lane 2 = negative control; lane 3 = positive control; blaOXA-23 was detected in lanes 4 (MDO-1), 6 (MDO-3), 10 (MDO-7), 11 (MDO-8), 14 (MDO-11), 16 (MDO-13), 19 (MDO-16), and 20 (MDO-17). Other isolates were negative for *blaOXA-23*. MDO = Manado.

**Figure 2:**
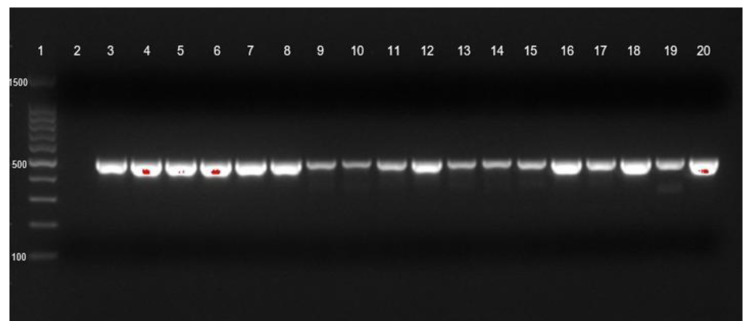
blaOXA-23 gene identification of CRAb-cc isolates from Dr. Saiful Anwar Hospital, Malang. Lane 1 = DNA ladder; lane 2 = negative control; lane 3 = positive control; blaOXA-23 was detected in lanes 4 to 20.

Bacterial DNA was isolated using the GeneAll^®^ ExgeneTM, Seoul, Republic of Korea. The DNA samples were stored at −20 °C. The detection of carbapenemase genes of seventy-three isolates of CRAB-cc were described elsewhere. Briefly, Ambler class A (bla*_KPC_*), Ambler class B (bla*_NDM_*), and Ambler class D (bla*_OXA-23_*) (Figure 1 and Figure 2) were detected by PCR [23]. The positive control was the CRAB-cc clinical isolates carrying the bla*_KPC_*, bla*_NDM_*, and bla*_OXA23_* confirmed by PCR in the previous study [19,31], and the negative control was a nuclease free-water. The primer sets and targeted genes are shown in Table 1.

### 2.4. Multi-Locus Variable-Number Tandem Repeat Analysis (MLVA) 

MLVA was carried out for 73 CRAB-cc isolates as previously described [34], using eight loci, including 3530, 3002, 2240, 1988, 0826, 0845, 2396, and 3468, as shown in the Table 2. PCR reactions for each locus VNTR program were as follows: initial denaturation of 5 min at 94 °C, 30 cycles of denaturation for 30 s at 94 °C, an annealing step for 30 s, an elongation step at 72 °C, and a final elongation step for 7 min at 72 °C. The number of repetitions for each allele was obtained by subtracting the flanking area and then dividing its value by the length of each repetition. The “failed” designation was given when no amplification or ambiguous amplicon patterns were observed repeatedly at a given locus, and the corresponding allele was indicated with a “-” symbol. Strains were compared using MLVAnet: http://mlva.u-psud.fr/MLVAnet/ (accessed on 1 February 2021) and were assigned into MLVA-8 complexes based on the criteria suggested by Pourcel C et al. [34].

### 2.5. Multilocus Sequence Typing (MLST)

The MLST was carried out based on the sequence analysis of the internal fragments of seven housekeeping genes: *cpn60* (60-kDa chaperonin), *fusA* (elongation factor EF-G), *gltA* (citrate synthase), *pyrG* (CTP synthase), *recA* (homologous recombination factor), *rplB* (50S ribosomal protein L2), and *rpoB* (RNA polymerase subunit B) adopted from the Institute Pasteur MLST method (Table 3) [27]. We selected randomly from two to four CRAb-cc isolates from each MLVA type (depends on the MLVA cluster size) for further analysis using MLST.

### 2.6. Clonal Complex (CC) Analysis

The CC analysis was performed using eBURST v.3 (http://eburst.mlst.net) (accessed on 24 December 2021) as previously described [35]. Eleven isolates of CRAB-cc analyzed by MLST were further identified for the CC analysis.

### 2.7. Phylogenetic Analysis

The phylogenetic analysis was generated from the eleven CRAB-cc isolates that were also investigated by MLST and CC analysis. The evolutionary relationship among isolates was built by combining sequences of the seven housekeeping genes, and it was then analyzed using MEGA X (Philadelphia, PA, USA) with the neighbor-joining method [36,37].

## 3. Results

A total of 73 CRAB-cc isolates were collected consecutively from clinical cultures (Manado: 30 isolates; Malang: 43 isolates). The CRAB-cc isolates were frequently found in lower-respiratory-tract specimens (42/73 (57.5%; 95% CI 45.4–69.0]), followed by pus (12/73 (16.4%; 95% CI 8.8–26.9]), blood (11/73 (15.1%; 95% CI 7.8–25.4]), and urine (8/73 (10.9%; 95% CI 4.9–20.5]). 

**Figure 3:**
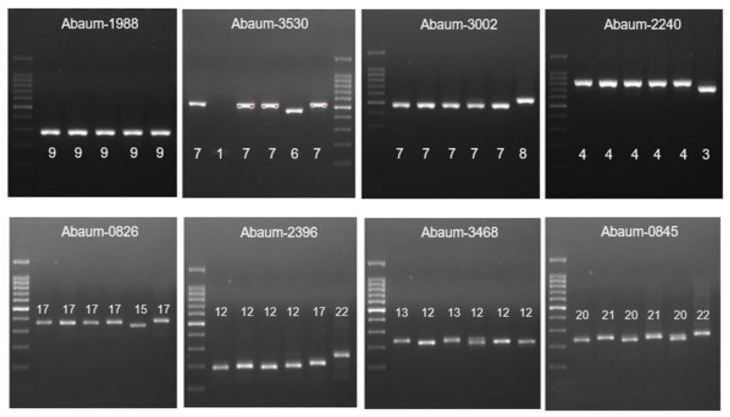
The MLVA typing of CRAb-cc isolates from Prof. Dr. R. D. Kandou Hospital, Manado. Lane 1 = DNA ladder; lane 2 = MDO-25; lane 3 = MDO-9; lane 4 = MDO-27; lane 5 = MDO-2; lane 6 = MDO-29; lane 7 = MDO-30*. * Abaum-1988: captured in another gel. Abaum-3530: the sample order is the same, but the DNA ladder is in lane 7.

**Figure 4:**
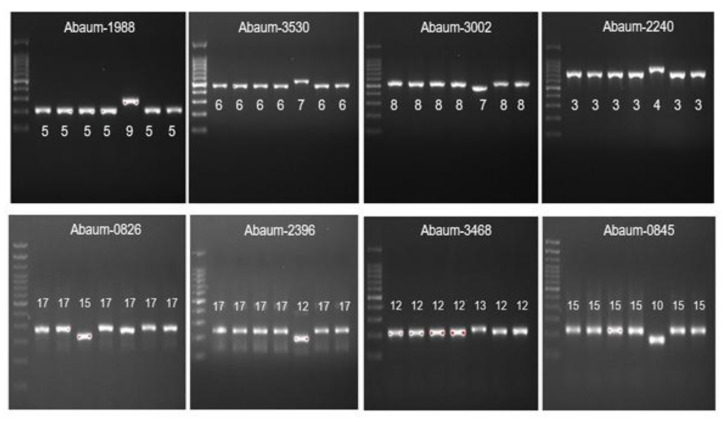
The MLVA typing of CRAb-cc isolates from Dr. Saiful Anwar Hospital, Malang. Lane 1 = DNA ladder; lane 2 = MLG-2; lane 3 = MLG-3; lane 4 = MLG-28; lane 5 = MLG-26; lane 6 = MLG-35; lane 7 = MLG-31; lane 8 = MLG-32.

The MLVA analysis was carried out to 73 CRAB-cc isolates generating six MLVA types (MT) as shown in the Table 4, Figure 3., and Figure 4. The most frequently found MT was MT2 obtained from lower-respiratory-tract specimen (24/35 (68.5%)), followed by blood (5/35 (14.3%)), pus (3/35 (8.6%)), and urine (3/35 (8.6%)). The MT2 cluster included 18 isolates from Malang and 17 isolates from Manado. In addition, we identified the MT3 as the second largest cluster predominantly found in Dr. Saiful Anwar Malang obtained from lower-respiratory-tract specimens (12/25 (48%)), followed by blood (6/25 (24%)), pus (4/25 (16%)), and urine (3/25 (12%)). The MT3 cluster included 21 CRAb-cc isolates from Dr. Saiful Anwar Hospital, Malang, and 4 CRAb-cc isolates from Prof. Dr. R. D. Kandou Hospital, Manado.

MLST was performed for eleven CRAb-cc isolates, consisting of one isolate from MT1, four isolates from MT2, three isolates from MT3, and one isolate of each of MT4, MT5, and MT6. The MLST analysis identified ST642 as the most frequently found sequence type among CRAb-cc isolates belonging to CC1 (Table 4) and corresponding to MT1, MT3, and MT4. The results of clonal complex analysis of eleven CRAb-cc isolates showed that CC1 and CC2 were predominantly found in Dr. Saiful Anwar Hospital, Malang, and Prof. Dr. R. D. Kandou Hospital, Manado, respectively (Table 5). Table 5 shows MLST and CC analysis of eleven CRAB-cc in Dr. Saiful Anwar Hospital, Malang, and Prof. Dr. R. D. Kandou Hospital, Manado compared to the MLVA types.

**Figure 5:**
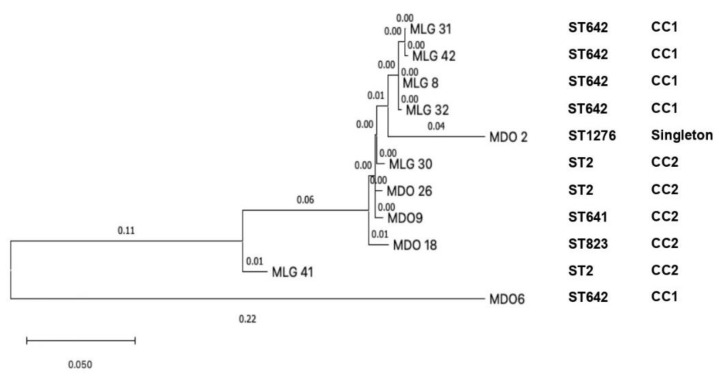
The phylogenetic tree of eleven clinical CRAb-cc isolates in Indonesia (MLG = Malang; MDO = Manado).

The phylogenetic analysis detected a close relationship among four isolates from Malang, including MLG-8, MLG-31, MLG-32, and MLG-42 (clade 1); in addition, MLG-30 from Malang was closely related to MDO-9 and MDO-26 from Manado (clade 2). The MDO-2 from Manado showed ST1276, which was independent and singleton; it is suggested that mutation had occurred. We found the MDO-6 strain corresponds to ST642 and CC1; it is suggestive that there are not many mutations in this strain [36]. Clade 1 and clade 2 were in concordance with CC1 and CC2, respectively (Figure 5). Another molecular epidemiology study on CRAb-cc was performed in a tertiary care hospital in Jakarta, Indonesia, performed by Saharman YR et al.; however, the phylogenetic analysis was not included [19]. 

## 4. Discussion

To the authors knowledge, the present study was the first clonality study of CRAb-cc clinical isolates in Malang and Manado. The present study showed that the CRAb-cc were frequently found in lower-respiratory-tract specimens. Abdulzahra AT et al. reported similar results in the hospital El-Kasr El-Aini (Cairo, Egypt). They found the predominant isolate of the *A. baumannii-calcoaceticus* complex in lower-respiratory-tract specimens [38].

Multi-locus variable-number tandem-repeat analysis showed two large MLVA types of CRAb-cc isolates in Dr. Saiful Anwar Hospital, Malang, designated MT2 and MT3, whereas one MLVA type (MT2) was predominantly found in Prof. Dr. R. D. Kandou Hospital, Manado. Similarly to other studies, we identified multiple MLVA types, including MT1, MT3, and MT4, within one sequence type (ST642). It is suggested that the three MLVA types are probably related.

The MLST and clonal complex analysis showed that ST642 CC1 corresponds to MT3, the most frequently found clone in this study. The predominance of ST642 CC1 in this study could not be determined to be a short-lived cluster of infections or a prolonged endemic because the baseline data are not available in both hospitals. The ST642 was also reported by another study in Indonesia conducted by Saharman YR et al., but not as the predominant sequence type among CRAb-cc strains in Dr. Cipto Mangunkusumo General Hospital, Jakarta, Indonesia [19], and in another study in Pakistan [39].

This study found five isolates consisting of four isolates from Malang and one isolate from Manado that were assigned to the same clone (ST642 CC1). It is suggested that the cross-transmission of ST642 CC1 may have occurred in Malang; however, the spreading of ST642 CC1 in Manado should be investigated with more samples. Further multicenter study involving more samples is required to get more insight into the molecular epidemiology of CRAb-cc strains in Indonesia.

ST2 CC2 was the second sequence type frequently found in this study, including two isolates from Malang and one isolate from Manado. ST2 was also found in Los Angeles [27], which might be associated with human mobility between countries or continents [24]. In addition, we detected three unique sequence types in this study, ST641, ST823, and ST1276, which were also found in Colombia [40], China [41], and Taiwan [42], respectively.

The phylogenetic tree showed the evolutionary relationship among CRAb-cc isolates obtained from Dr. Saiful Anwar Hospital in Malang (clade 1), and that among isolates from Dr. Saiful Anwar Hospital in Malang and Prof. Dr. R. D. Kandou Hospital in Manado (clade 2). A mathematical model study reported that the evolution of multidrug-resistant organisms is more likely to occur when the bacteria are exposed to the antibiotics. The kinds of antibiotics used in the population might be associated with a genome that is ready to evolve [21,43,44,45]. However, more isolates are required to identify the phylogenetics among CRAB-cc isolates in the two tertiary care hospitals in this study.

The reservoir of CRAB-cc isolates in this study is unknown. The pathogen could survive in the adverse environmental conditions, fostering its persistence and spread in the hospital environment through the hands of the hospital staff, respiratory therapy equipment, tap water, soap dispensers, food (including hospital food), patient beds (mattresses, pillows, bed curtains and blankets), infusion pumps, stainless steel trolleys, door handles, and telephone handles [46,47]. Nevertheless, further investigation is required to discover the source of transmission of CRAB-cc isolates in Dr. Saiful Anwar Hospital and Prof. Dr. R. D. Kandou Hospital.

The present study has certain limitations. First, this study was conducted in two tertiary care hospitals in Indonesia; therefore, it cannot represent national data yet. However, it could be used as a point reference of the molecular epidemiology of CRAB-cc strains in Indonesia. Second, we did not perform the MLST, clonal complex analysis, and phylogenetic analysis for all samples due to limited resources; therefore, the clonal relatedness of all isolates obtained from clinical specimens in Dr. Saiful Anwar Hospital and Prof. Dr. R. D. Kandou Hospital could not be provided.

## 5. Conclusions

The ST642-CC1 was detected as the most common clone of CRAb-cc in Dr. Saiful Anwar Hospital in Malang, In addition, this study detected one clade of CRAb-cc isolates designated as clade 2 (ST2-CC2) that consisted of one CRAb-cc isolate from Dr. Saiful Anwar Hospital (Malang) and one CRAb-cc isolate from Prof. Dr. R. D. Kandou Hospital (Manado), representing a clonal relatedness between CRAb-cc in Dr. Saiful Anwar Hospital (Malang) and Prof. Dr. R. D. Kandou Hospital (Manado). Human mobility and antibiotic prescription behavior may be associated with the emerging of a new clade of MDRO in a hospital setting. A national surveillance system is required to have more insight regarding the molecular epidemiology of CRAB-cc in Indonesian hospitals and to develop a strategy to control the spread of CRAB-cc in Indonesian hospitals.

## Figures and Tables

**Table 1:** Gene primers used for PCR amplification of antibiotic resistance genes in clinical CRAB-cc isolates.

Targeted Gene	Primer Designation	Amplicon Size	Tm °C	Primer Sequence
bla*_KPC_*	bla*_KPC_*-F bla*_KPC_*-R	798 bp	58	CGTCTAGTTCTGCTGTCTTG CTTGTCATCCTTGTTAGGCG
bla*_NDM_*	bla*_NDM_*-F bla*_NDM_*-R	621 bp	65	GGTTTGGCGATCTGGTTTTC CGGAATGGCTCATCACGATC
bla*_OXA23_*	bla*_OXA23_*-F bla*_OXA23_*-R	501 bp	52	GATCGGATTGGAGAACCAGA ATTCTGACCGCATTTCCAT

Abbreviations: bla*_KPC_*, *K. pneumonia carbapenemase*; bla*_NDM_*, *New-Delhi metallo-b-lactamase*; bla*_OXA23_*, oxacillinase-23; Tm, temperature of melting.

**Table 2:** Primers, PCR conditions, and characteristics of VNTRs analyzed.

VNTR Marker	Primer Designation	Primer Sequence (5′→3′)	Annealing Temperature (°C)	Repeat Size (bp)	Size of Flanking Regions
Abaum-1988	*Abaum-1988-F*	GGCAAGGCATGCTCAAGGGCC	55	26	77
	*Abaum-1988-R*	CAGTAGACTGCTGGTTAATGAG	55		
Abaum-3530	*Abaum-3530-F*	TGCAACCGGTATTCTAGGAAC	55	60	121
	*Abaum-3530-R*	CCTTGAACAACATCGATTACTGGA	55		
Abaum-3002	*Abaum-3002-F*	GACTGAAGCAAGACTAAAACGT	55	57	121
	*Abaum-3002-R*	TCTGGGCAGCTTCTTCTTGAGC	55		
Abaum-2240	*Abaum-2240-F*	CCCGCAGTACATCATGGTTC	55	99	494
	*Abaum-2240-R*	AGAACATGTATACGCAACTG	55		
Abaum-0826	*Abaum-0826-F*	TGACTACTGAAACAGTTTTTG	50	9	208
	*Abaum-0826-R*	ATGATTGTACCGAGTAAAAGA	50		
Abaum-2396	*Abaum-2396-F*	CAAGTCCAATCAACTCATGATG	55	6	105
	*Abaum-2396-R*	CTCCTGTAAGTGCTGTTCAGCC	55		
Abaum-3468	*Abaum-3468-F*	CAGAAGTCACTGCATCTGCAAC	55	6	147
	*Abaum-3468-R*	CGGTTGAAATTTTTTATAATGAAG	55		
Abaum-0845	*Abaum-0845-F*	AATTTTAATTCCAAATTGCTCC	50	7	105
	*Abaum-0845-R*	ACTTAAAATCGCATTTTTATCA	50		

Abbreviations: VNTR, variable-number tandem-repeat; Abaum, *A. baumannii.*

**Table 3:** Gene primers used for the detection of housekeeping genes by PCR in genes in CRAB-cc isolates in MLST studies.

Gene	Primer Suquence	Amplicon Size
*cpn60-F*	ACTGTACTTGCTCAAGC	405 bp
*cpn60-R*	TTCAGCGATGATAAGAAGTGG	
*fusA-F*	ATCGGTATTTCTGCKCACATYGAT	633 bp
*fusA-R*	CCAACATACKYTGWACACCTTTGTT	
*gltA-F*	AATTTACAGTGGCACATTAGGTCCC	483 bp
*gltA-R*	GCAGAGATACCAGCAGAGATACACG	
*pyrG-F*	GGTGTTGTTTCATCACTAGGWAAAGG	297 bp
*pyrG-R*	ATAAATGGTAAAGAYTCGATRTCACCMA	
*recA-F*	CCTGAATCTTCYGGTAAAAC	372 bp
*recA-R*	GTTTCTGGGCTGCCAAACATTAC	
*rplB-F*	GTAGAGCGTATTGAATACGATCCTAACC	330 bp
*rplB-R*	CACCACCACCRTGYGGGTGATC	
*rpoB-F*	GGTCCTGGTGGTTTAACACG	456 bp
*rpoB-R*	CGAATAACGATACGAGAAGCA	

Abbreviations: cpn60, 60 kDa chaperonin; fusA, elongation factor EF-G; gltA, citrate synthase; pyrG, CTP synthase; recA, homologous recombination factor; rplB, 50S ribosomal protein L2; rpoB, RNA polymerase subunit B.

**Table 4:** MLVA typing results of CRAb-cc isolates obtained from Dr. Saiful Anwar Hospital, Malang, and Prof. Dr. R. D. Kandou Hospital, Manado, Indonesia (*n* = 73).

No.	MT	MLVA Loci								Number of Isolates (%)	
		Abaum-1988	Abaum-3530	Abaum-3002	Abaum-2240	Abaum-0826	Abaum-2396	Abaum-3468	Abaum-0845	Dr. Saiful Anwar Hospital, Malang	Prof. Dr. R. D. Kandou Hospital, Manado
1.	MT1	5	6	7	3	15	17	12	20	4 (9.3%)	
2.	MT2	9	7	7	4	17	12	12	20	18 (41.9%)	17 (56.6%)
3.	MT3	5	6	8	3	17	22	12	22	21 (48.8%)	4 (13.3%)
3.	MT4	5	1	7	3	17	12	12	22		5 (16.7%)
5.	MT5	8	6	8	3	-	17	13	20		2 (6.7%)
6.	MT6	9	1	-	-	-	17	9	20		2 (6.7%)

Abbreviations: MLVA, multi-locus variable-number tandem-repeat analysis; MT, MLVA type.

**Table 5:** MLST and CC analyses of eleven CRAB-cc in Dr. Saiful Anwar Hospital, Malang, and Prof. Dr. R. D. Kandou Hospital, Manado compared to the MLVA types.

No.	MT	Sample Code	Locus Sequence and Allele Number							ST	CC
			*cpn60*	*fusA*	*gltA*	*pyrG*	*recA*	*rpIB*	*rpoB*		
1.	MT1	MLG-42	1	1	1	1	9	1	1	642	1
2.	MT2 MT2 MT2 MT2	MLG-30	2	2	2	2	2	2	2	2	2
3.		MLG-41	2	2	2	2	2	2	2	2	2
4.		MDO-18	2	125	2	2	2	2	2	823	2
5.		MDO-26	2	2	2	2	2	2	2	2	2
6.	MT3 MT3 MT3	MLG-8	1	1	1	1	9	1	1	642	1
7.		MLG-31	1	1	1	1	9	1	1	642	1
8.		MLG-32	1	1	1	1	9	1	1	642	1
9.	MT4	MDO-6	382	267	14	188	9	17	1	642	1
10.	MT5	MDO-2	192	26	91	14	192	16	47	1276	singleton
11.	MT6	MDO-9	2	1	1	2	68	2	201	641	2

Abbreviations: MLVA, multi-locus variable-number tandem-repeat analysis; MT, MLVA types; MLST, multi-locus sequence type; ST, sequence type; CC, clonal complex; MLG, Malang; MDO, Manado.

## Data Availability

The data of this study are available from the corresponding author on request.
